# Comparison of unitary exocytic events in pituitary lactotrophs and in astrocytes: modeling the discrete open fusion-pore states

**DOI:** 10.3389/fncel.2013.00033

**Published:** 2013-04-04

**Authors:** Doron Kabaso, Jernej Jorgačevski, Ana I. Calejo, Ajda Flašker, Alenka Guček, Marko Kreft, Robert Zorec

**Affiliations:** ^1^Laboratory of Biophysics, Faculty of Electrical Engineering, University of LjubljanaLjubljana, Slovenia; ^2^Laboratory of Neuroendocrinology-Molecular Cell Physiology, Faculty of Medicine, Institute of Pathophysiology, University of LjubljanaLjubljana, Slovenia; ^3^Celica Biomedical CenterLjubljana, Slovenia; ^4^Departamento de Biologia e CESAM, Universidade de AveiroAveiro, Portugal; ^5^Department of Biology, Biotechnical Faculty, University of LjubljanaLjubljana, Slovenia

**Keywords:** capacitance measurements, equivalent circuit, transient fusion-pore, modeling, astrocytes

## Abstract

In regulated exocytosis the merger between the vesicle and the plasma membranes leads to the formation of an aqueous channel (a fusion-pore), through which vesicular secretions exit into the extracellular space. A fusion pore was thought to be a short-lived intermediate preceding full-fusion of the vesicle and the plasma membranes (full-fusion exocytosis). However, transient exocytic events were also observed, where the fusion-pore opens and closes, repetitively. Here we asked whether there are different discrete states of the open fusion-pore. Unitary exocytic events were recorded by the high-resolution cell-attached patch-clamp method in pituitary lactotrophs and brain astrocytes. We monitored reversible unitary exocytic events, characterized by an on-step, which is followed by an off-step in membrane capacitance (*C*_*m*_), a parameter linearly related to the membrane area. The results revealed three categories of reversible exocytic events (transient fusion-pore openings), which do not end with the complete integration of the vesicle membrane into the plasma membrane. These were categorized according to the observed differences in the amplitude and sign of the change in the real (*Re*) parts of the admittance signals: in case I events (*Re* ≈ 0) fusion pores are relatively wide; in case II (*Re* > 0) and case III (*Re* < 0) events fusion pores are relatively narrow. We show that case III events are more likely to occur for small vesicles, whereas, case II events are more likely to occur for larger vesicles. Case III events were considerably more frequent in astrocytes than in lactotrophs.

## Introduction

In regulated exocytosis the fusion between the vesicle and the plasma membrane is important not only for the secretion of signaling molecules, such as hormones and transmitters, but is also key for the membrane recycling and for the translocation of membrane receptors and other proteins to the plasma membrane (White, 1992; Jahn et al., 2003; Vardjan et al., 2009). The membrane fusion process is an energetically unfavorable event, since it consists of bringing close to each other negatively charged opposing membranes, and involves the bending of a lipid-bilayer into a highly curved structure, which is assembled by different proteins and lipids (Kozlov and Markin, 1983). Following the merger of the two membranes, an important step in regulated exocytosis is the formation of a fusion-pore, an aqueous channel connecting the vesicle lumen and the exterior of the cell (reviewed in Chernomordik and Kozlov, 2008).

The nature of this structure can be studied by the electrophysiological patch-clamp membrane capacitance (*C*_*m*_) technique, which allows monitoring interactions of a single vesicle with the plasma membrane (Neher and Marty, 1982; Lindau and Neher, 1988; Lollike et al., 1995), and the dynamics of an individual fusion-pore. In addition to simple discrete step increases in *C*_*m*_, which were considered to represent full-fusion exocytic events (Neher and Marty, 1982), some of the unitary exocytic events are transient in nature, characterized by repetitive discrete on-steps (increases) followed by an equal amplitude off-steps (decreases) in *C*_*m*_. The increment and the ensuing decrement step in *C*_*m*_ are considered to be due to a transient fusion-pore opening of a fused vesicle (Heuser and Reese, 1973; Alvarez de Toledo et al., 1993; Vardjan et al., 2007). These events were also termed transient or reversible exocytic events, also »kiss-and-run« exocytosis. Transient fusion-pore openings are considered physiologically relevant, since the fusion-pore is not formed upon each round of exocytosis (Ceccarelli et al., 1972; Kozlov and Markin, 1983). Moreover, a narrow fusion-pore, once it is established, appears to be energetically relatively favorable (Jorgačevski et al., 2010, 2011).

These conclusions were made on the basis of monitoring repetitive unitary exocytic events in which the narrowness of the fusion-pore can be determined from optical and electrophysiological measurements (Vardjan et al., 2007; Jorgačevski et al., 2008). In electrophysiological measurements specifically, by determining fusion-pore conductance (*G*_*p*_) and vesicle capacitance (*C*_*v*_) from admittance measurements (Lindau, 1991; Lollike and Lindau, 1999). In these recordings, changes observed in the imaginary (Δ*Im*) and in the real (Δ*Re*) parts of admittance signals reflect changes in *C*_*v*_ and *G*_*p*_, which can be used to determine vesicle diameter and fusion pore diameter (Lindau, 1991; Rosenboom and Lindau, 1994). Occasionally, the admittance traces associated with a transient fusion-pore opening exhibit an incremental or a decremental cross-talk in the *Re* signal (Breckenridge and Almers, 1987; Lindau, 1991; Henkel et al., 2000). However, the underlying mechanisms responsible for these non-zero projections in the *Re* signal are not fully understood.

In previous studies, the equivalent circuit of the fusion pore was reported (Lindau and Neher, 1988; Scepek and Lindau, 1993; Lollike et al., 1995). It was shown analytically that the incremental cross-talk projection on the *Re* signal could be due to the fusion-pore opening devoid of complete vesicle membrane integration into the plasma membrane (Lindau, 1991; Henkel et al., 2000). By studying pituitary lactotrophs, an ideal cell preparation to study secretory activity at the single vesicle level (Stenovec et al., 2004; Vardjan et al., 2007), and astrocytes which release gliotransmitters (Parpura et al., 1994) by likely employing regulated exocytosis (Parpura and Zorec, 2010), we compared the properties of unitary exocytic events. Using equivalent circuit analysis, we here demonstrate that the decremental cross-talk projection on the *Re* signal depends on the *G*_*p*_ as well as on the size of the fused vesicle. Moreover, these results indicate the existence of a very narrow, nearly closed, open fusion-pore state in pituitary lactotrophs and in astrocytes.

## Materials and methods

### Cell cultures

Primary lactotroph and astrocyte cultures were prepared from adult male (lactotrophs) and 2–3 days old female Wistar rats as described previously (Schwartz and Wilson, 1992; Ben-Tabou et al., 1994; Jorgačevski et al., 2008). After the isolation we plated cells on poly-L-lysine-coated coverslips and maintained them in high-glucose DMEM (Invitrogen) medium, supplemented with 10% newborn calf serum and 2 mM L-glutamine in an atmosphere of humidified air (95%) and CO_2_ (5%). We cared for the experimental animals in accordance with the International Guiding Principles for Biomedical Research Involving animals, developed by the Council for International Organizations of Medical Sciences, and the Directive on Conditions for Issue of License for Animal Experiments for Scientific Research Purposes (Official Gazette of the Republic of Slovenia 40/85 and 22/87). The procedures using animals were approved by the Veterinary Administration of the Republic of Slovenia (approval no. 34401-29/2009/2).

### Electrophysiology

Cell-attached capacitance measurements on isolated rat lactotrophs and astrocytes were performed with a dual-phase lock-in patch-clamp amplifier (SWAM IIC and SWAM CELL, Celica, Ljubljana, Slovenia) as described (Kreft and Zorec, 1997; Vardjan et al., 2007; Jorgačevski et al., 2010). Briefly, a sine wave voltage (1591 or 6400 Hz, 111 mV) was applied to the pipette, while holding the pipette potential at 0 mV. The phase of the lock-in amplifier was adjusted to nullify the changes in *Re*. A 10 fF calibration pulse was manually generated every 10 s to ensure correct phase angle settings. We used thick-walled, fire polished glass pipettes, which were heavily coated with a resin (Sylgard®184) and had a resistance of 2–5 MΩ.

### The equivalent circuit of a transient fusion pore

**Figure 1:**
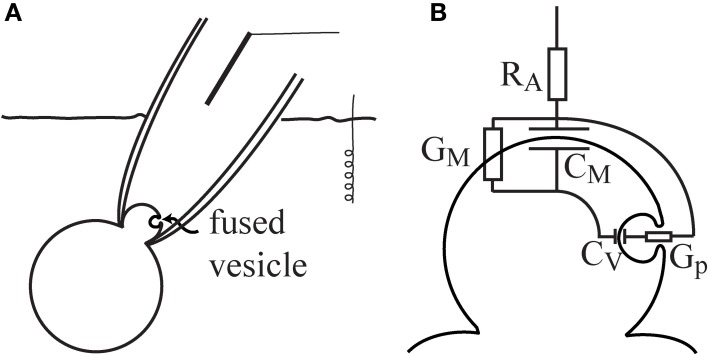
**The equivalent circuit of a patched membrane with a fused vesicle.** The cell-attached patch-clamp configuration enables the detection of vesicle fusion in the patched membrane region **(A)**. In the equivalent circuit, the fusion of a vesicle is considered in parallel to the patch membrane **(B)**. Note that *R*_*A*_ is the access resistance, *C*_*m*_ is the whole-cell membrane capacitance, *G*_*M*_ is the membrane conductance, *C*_*v*_ is the vesicle membrane capacitance, and *G*_*p*_ is the fusion-pore conductance.

A patch-clamp configuration could be approximated by the series combination of the membrane and the access resistance (*R*_*A*_) of the pipette tip through the patch (Figure 1A). The membrane included a parallel setup of the whole-cell *C*_*m*_ and the membrane conductance (*G*_*M*_). When a small vesicle fused with the patch region, the patch-clamp system could detect the fusion event by the observed changes in the admittance measurement. It has been previously confirmed that the fusion of a vesicle was accompanied by an increase in the measured *C*_*m*_ (Neher and Marty, 1982). The equivalent circuit of the patch-configuration with a fused vesicle is shown in Figure 1B. The vesicle *C*_*m*_ is denoted by *C*_*v*_, and the fusion-pore conductance is denoted by *G*_*p*_.

In the present analysis, all the parameters except *G*_*p*_ were held constant. It was assumed that *C*_*v*_ << *C*_*m*_ or ω*C*_*v*_ << 1/*R*_*A*_, which are reasonable assumptions, since the vesicle *C*_*m*_ (vesicle surface area) is considerably smaller than the cell *C*_*m*_ (cell surface area), and the patch resistance is considerably greater than the capacitor load of the vesicle (ω*C*_*v*_). The admittance change (Δ*Y*) describes the change in the admittance between the open and nearly closed state of the fusion pore. According to the equivalent circuit, the admittance difference is (Lindau, 1991):

(1) $$\Delta Y=T^{2}(\omega )\text{​}\left(\frac{{\left(\omega C_{v}\right)}^{2}/G_{p}}{1+{\left(\omega C_{v}/G_{p}\right)}^{2}}+i\frac{\omega C_{v}}{1+{\left(\omega C_{v}/G_{p}\right)}^{2}}\right),$$

where *T*^2^(ω) stands for the factor *T*^2^ (ω) = 1/(1 + *R*_*A*_*G*_*M*_ + *i*ω*C*_*m*_*R*_*A*_)^2^ = |*T*(ω)|^2^ · *e*^*i*θ^, and *i* is $\sqrt{-1}$. The admittance change (Δ*Y*) is an imaginary number, in which the first term in the parenthesis is the Δ*Re*, and the second term in the parenthesis is the Δ*Im*. For the sake of simplicity, the fusion pore state is denoted as fully open or incompletely open. In the fully open state, the *G*_*p*_ is infinite (*G*_*p*_ → ∞), and the Δ*Re* in Equation (1) vanishes, which gives Δ*Y* = *iT*^2^ (ω)ω*C*_*v*_. On the other hand, when there is incomplete fusion, the *G*_*p*_ can be on the same scale as ω*C*_*v*_. As a result, both the Δ*Re* and Δ*Im* have a finite value, which can be used for the calculation of the unknown *G*_*p*_ (Breckenridge and Almers, 1987). The *C*_*v*_ and the *G*_*p*_ can be obtained from the real and imaginary parts (Lindau, 1991; Lollike and Lindau, 1999), as follows:

(2) $$\begin{array}{l} C_{v}=\frac{\Delta Re^{2}+\Delta Im^{2}}{\Delta Im}/\omega \\ G_{p}=\frac{\Delta Re^{2}+\Delta Im^{2}}{\Delta Re}. \end{array}$$

## Results

### Admittance measurements of the three cases of transient exocytic events

**Figure 2:**
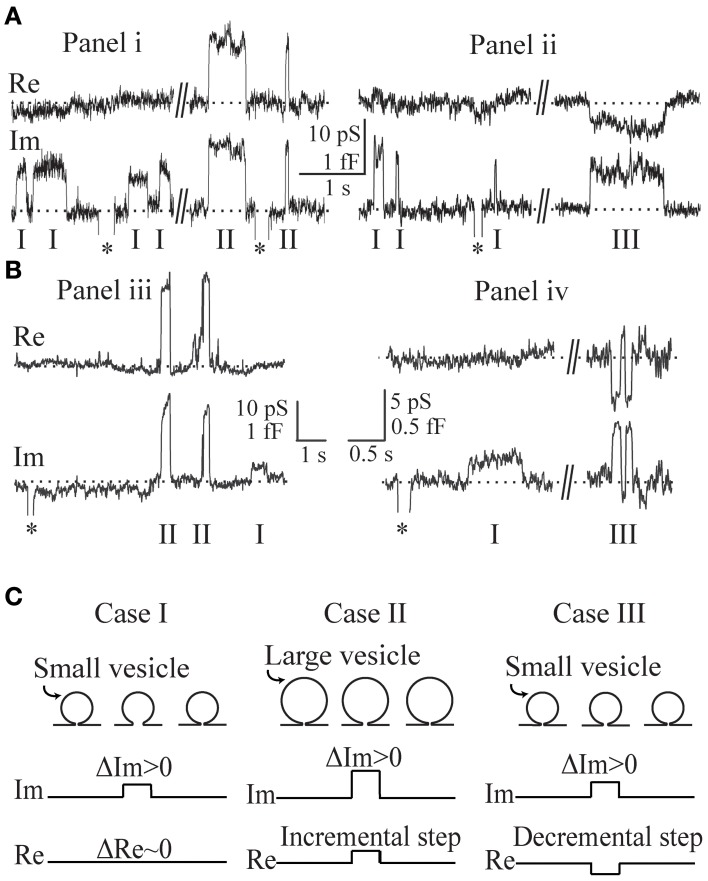
**Representative admittance measurements recorded in lactotrophs (Ai,ii) and astrocytes (Biii,iv) revealing a zero projection (case I), an incremental projection (case II), and a decremental projection (case III), on the *Re* part of the admittance signal.** The asterisks in these recordings indicate calibration pulses. The recordings in **(Ai,ii)** and **(Biii,iv)** are recorded in the same patch of a lactotroph and an astrocyte, respectively. By incorporating the estimated Δ*Im* and Δ*Re* into the Equation (2), the corresponding vesicle capacitance amplitudes are determined for each event from the admittance records. For the recording in lactotrophs **(Ai,ii)**, the vesicle capacitance of case I, case II, and case III, is 0.9 fF, 4.0 fF, and 1.2 fF, and for the astrocytes **(Biii,iv)**, it is 0.3 fF, 1.6 fF, and 0.6 fF, respectively. Note the larger vesicle capacitance of case II when compared to case I. A schematic representation of the three cases as pre-fused vesicles with an initial narrow fusion-pore **(C)**.

The admittance measurements of repetitive opening and closure of fusion-pores were obtained in lactotrophs (Figures 2Ai,ii) and in astrocytes (Figures 2Biii,iv), where representative transient exocytic events are shown, denoted as cases I, II, and III on Figure 2. The asterisks in these recordings indicate calibration pulses which were used to adjust the phase of the lock-in amplifier. The events in panels Figures 2A,B were recorded in the same membrane patch of a lactotroph and an astrocyte, respectively. Using Equation (2), the corresponding vesicle surface area in fF is calculated by incorporating Δ*Im* and Δ*Re* estimated for each event from the admittance records. On Figure 2A (recorded in lactotrophs) the *C*_*v*_ in case I, case II, and case III, was 0.9, 4, and 1.2 fF, respectively. Whereas, on Figure 2B, where representative recordings in astrocytes are shown, the *C*_*v*_ was in case I (0.3 fF), case II (1.6 fF), and case III (0.6 fF). One can note that the amplitude in *Im*, reflecting *C*_*v*_, appears larger in events of case II in comparison with case I events. Case I events exhibit an incremental change in *Im* trace and are devoid of projection on the *Re* trace. In case II events and increment in *Im* trace is associated with an incremental projection on the *Re* trace, whereas, in case III events an increment in *Im* trace is associated with a decremental projection on the *Re* trace. In Figure 2C the three cases of transient exocytic events are diagrammatically presented as pre-fused vesicles with an initial narrow fusion-pore (narrower than the detection limit of the recording system), which can reversibly widen to a larger diameter—a state that can be detected electrophysiologically.

**Figure 3:**
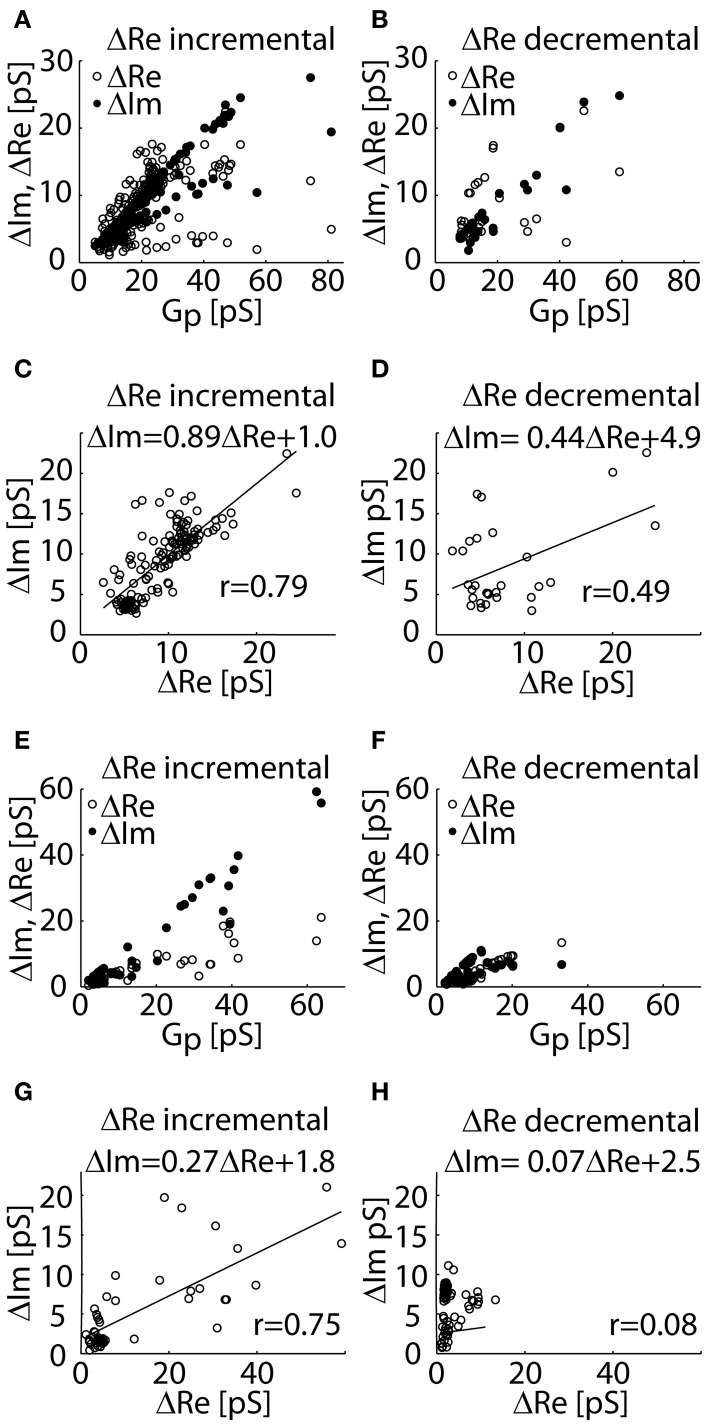
**The experimental datasets of Δ*Re* and Δ*Im* of admittance measurements recorded in lactotrophs (A–D) and astrocytes (E–H) incremental (case II) and decremental (case III) Δ*Re* projections.** The distributions of Δ*Re* and Δ*Im* as a function of *G*_*p*_ for the datasets of incremental Δ*Re* **(A,E)** and for the decremental Δ*Re* **(B,F)** datasets. A scatter plot of Δ*Im* against Δ*Re* reveals a strong linear relationship in the incremental Δ*Re* datasets of projections **(C,E)**, and a weak linear correlation in the dataset of decremental Δ*Re* projections **(D,H)**.

The experimental datasets used in our analysis, include Δ*Re* and Δ*Im* of cases I–III obtained in lactotrophs (Figures 3A–D) and astrocytes (Figures 3E–H). The *G*_*p*_ of each event is calculated from Δ*Re* and Δ*Im* of cases II (Δ*Re* incremental) and III (Δ*Re* decremental). Figures 3A–H shows scatter plots of Δ*Re* and Δ*Im* as a function of *Gp*. The relationships between Δ*Im* versus Δ*Re* are plotted for the datasets of incremental and decremental Δ*Re* projections. A strong linear relationship (*r* = 0.79 in Figure 3C; *r* = 0.75 in Figure 3G) was revealed between Δ*Im* and Δ*Re* in the incremental Δ*Re* projection group (Figures 3C,G). The slope of this relationship in astrocytes is smaller than one (Figure 3G), however that in lactotrophs is close to unity (Figure 3C), suggesting that Δ*Re* and Δ*Im* are of similar size. The non-zero Δ*Re* can be attributed to the incomplete opening of the fusion-pore, which is accompanied by the increased fusion-pore resistance, whereas, the fully open fusion-pore exhibit a negligible resistance. On the other hand, in the decremental Δ*Re* projection datasets, a weak correlation between Δ*Im* and Δ*Re* was revealed (*r* = 0.49 in Figure 3D; *r* = 0.08 in Figure 3H). We noted that the datasets of decremental *Re* projections are clustered more in the range of small *G*_*p*_ (Figures 3B,F).

### Reproducing the three cases of admittance measurements

Next, we considered the mechanisms responsible for the incremental and decremental Δ*Re* projections in the admittance measurements of the two cell types. According to the equivalent electrical circuit, the change in the real (Δ*Re*) and imaginary (Δ*Im*) parts of admittance signals can be employed for the calculation of *C*_*v*_ and *G*_*p*_ (Lindau, 1991; Lollike and Lindau, 1999).

**Figure 4:**
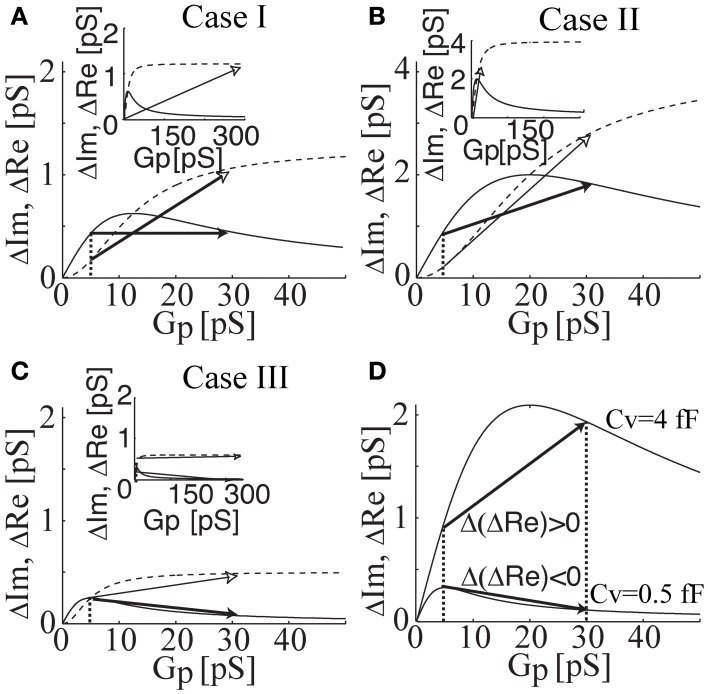
**The relationships of Δ*Re* (solid lines) and Δ*Im* (dahsed lines) as a function of *G*_*p*_ calculated for intermediate (*C*_*v*_ = 1.2 fF; case I), large (*C*_*v*_ = 4 fF; case II), and small (*C*_*v*_ = 0.5 fF; case III) vesicle size.** Note that the change in Δ*Re* and Δ*Im* are drawn by a full and an open arrow, respectively. The zero projection on the *Re* part is reproduced by a change from a fully closed state to a fully open fusion-pore state (see inset) or by a change from a nearly closed state to an incompletely open fusion-pore state **(A)**. The incremental projection in case II is reproduced by a change from nearly closed to incompletely open state **(B)**. The decremental projection in case III can be reproduced, only when the pre-fused state is nearly closed **(C)**. In addition, the change in Δ*Re* can be considerably larger than the change in Δ*Im* (see inset). Overlay of Δ*Re* (*Gp*) of cases I and III reveals the effect of vesicle size on the projection type **(D)**. By having the same pre-fused state (i.e., the same fusion pore conductance), the incomplete fusion-pore opening of a large vesicle size leads to an incremental projection, whereas, the same incomplete opening of a small vesicle causes a decremental projection in *Re*.

In Figure 4, Δ*Re* and Δ*Im* are plotted as a function of *G*_*p*_ calculated for intermediate (*C*_*v*_ = 1.2 fF), large (*C*_*v*_ = 4 fF), and small (*C*_*v*_ = 0.5 fF) vesicles, representing events of case I, II, and III, respectively. The fact that in case I there is no projection on the *Re* trace can be reproduced by a change from a fully closed fusion-pore state (*G*_*p*_ = 0 pS) to a fully open fusion-pore state (e.g., *G*_*p*_ > 500 pS in lactotrophs and *G*_*p*_ > 70 pS in astrocytes) or by a change from a nearly closed fusion-pore state (e.g., *G*_*p*_ = 5 pS in lactotrophs and *G*_*p*_ = 2 pS in astrocytes) to an incompletely open fusion-pore state (e.g., *G*_*p*_ = 30 pS) (Figure 4A). The positive projection in case II can be due to fusion-pore opening from a fully closed state, or a nearly closed state, to an incompletely open state (Figure 4B). On the other hand, the negative projection in case III can be reproduced only when the pre-fused state is nearly closed (Figure 4C), which suggests the possible existence of a nearly closed state of the fusion-pore. The underlying hypothesis for the different transient exocytic event cases is that the incremental and decremental projections to the *Re* trace are due to differences in the fused vesicle size and its nearly closed fusion-pore state. According to the presented equivalent circuit, small vesicles will tend to exhibit decremental Δ*Re* projections when their initial fused state is nearly closed. In Figure 4D, the relationships of Δ*Re* as a function of *G*_*p*_ of a small and a large vesicle are demonstrated. The overlay of the two relationships reveals that the maximum of Δ*Re* is at a lower *G*_*p*_ (*G*_*p*_ = 5 pS) for the small vesicle than the counterpart (*G*_*p*_ = 20 pS) in the case of the large vesicle. Assuming that initial vesicle fusion status arises from the same nearly closed state (e.g., *G*_*p*_ = 5 pS), the incomplete opening of the fusion pore would lead to a positive projection in the case of the large vesicle and a decremental projection in the Δ*Re* in the case of the small vesicle (Figure 4D).

## Discussion

In the present paper, we analyzed the discrete open fusion-pore states as well as the conditions under which these states existed. Three different cases are categorized, in which the transitions between discrete states of the fusion-pore do not end with the complete fusion (i.e., exocytosis) of a vesicle and the plasma membrane.

These cases are evident from the changes in the real (Δ*Re*) and imaginary (Δ*Im*) parts of admittance measurements (Figure 2). In the first case (denoted as case I), the event is characterized by a step increase in Δ*Im* and an approximately zero Δ*Re*. In the second case (denoted as case II), both Δ*Im* and Δ*Re* are exhibiting a step increase. The third case (denoted as case III) has an incremental Δ*Im* and a decremental Δ*Re*. The underlying assumption of our model is that the non-zero Δ*Re* is due to an incomplete vesicle fusion-pore opening, and that the initial status of the fused vesicle may exhibit a non-zero *G*_*p*_ (Figure 2). The equivalent circuit of a patch-clamp configuration was constructed, in which the fused vesicle is considered in parallel to the cell plasma membrane (Figure 1). The fused vesicle is assumed to be connected to the cell membrane via an aqueous channel (the fusion-pore). The *C*_*v*_ and the *G*_*p*_ are derived from the imaginary (Δ*Im*) and real (Δ*Re*) parts of the admittance measurements. The experimental data of Δ*Re* and Δ*Im* are obtained from admittance measurements in lactotrophs and astrocytes (Figure 2). In case II, a strong linear relationship between Δ*Re* and Δ*Im* suggests that the fusion-pore opening of a vesicle is incomplete (Figures 3C,G). In case III, there is a weak correlation between Δ*Re* and Δ*Im* (Figures 3D,H). According to the relationships of Δ*Re* and Δ*Im* as a function of *G*_*p*_, it is demonstrated that the decremental *Re* projection is more likely to occur for small vesicles (Figure 4C). Finally, the present calculations reveal that while the incomplete opening of the fusion-pore may be accompanied by the same change in fusion-pore conductance, the resulted projection is predicted to be decremental for the relatively small vesicle and incremental for the large vesicle (Figure 4D).

The high bending energy during the formation of a fusion-pore can be overcome by the assembly of curvature membrane constituents (proteins and lipids) (Kozlov and Markin, 1983; Jorgačevski et al., 2010; Kabaso et al., 2012; Jesenek et al., 2012). The stability of the fusion-pore of a fused vesicle can be due to anionic lipids of negative spontaneous curvature, modulating the formation of the fusion pore (Coorssen and Rand, 1990; Chen and Rand, 1997; Churchward et al., 2008; Rituper et al., 2012). It is then possible that the density of these curvature membrane constituents can affect the *G*_*p*_ of the nearly closed fusion-pore state as well as the projection type and amplitude. The possible incomplete fusion-pore state opens a communication venue, in which the passage of small molecules such as ions may be facilitated continuously through the narrow pore. However, larger molecular weight molecules are unable to exit the narrow fusion pore.

What appears interesting is that in both cell types, electrically excitable (pituitary lactotrophs) and electrically non-excitable (brain astrocytes), fusion-pore properties appear to be shared. While vesicles in the lactotrophs exhibit larger diameters and are therefore more accessible to experimentation (Stenovec et al., 2004; Vardjan et al., 2007), secretory vesicles in astrocytes appear to exhibit relatively large and relatively small diameters. The latter ones can be revealed by the higher-resolution cell-attached patch-clamp measurements (Kreft and Zorec, 1997). Interestingly, it is the smaller ones that exhibit fusion-pores with extremely narrow fusion-pore diameters (Figure 4). The probability of observing an increment in *Im* trace, associated with a decremental change in *Re* trace, indicates that a relatively large fraction of vesicles, which are already fused with the plasma membrane, exhibit a very narrow fusion pore. These may pass protons as has been reported previously by using a pH-sensitive vesicle luminal fluorophore in lactotrophs (Vardjan et al., 2007) and in astrocytes (Malarkey and Parpura, 2011). However the relatively large abundance of these events recorded in astrocytes, may not mean that fusion-pore openings mediate a productive release of gliotransmitters. A *G*_*p*_ of less than 5 pS means that the fusion pore diameter is less than 0.2 nm, too narrow to pass even glutamate or acetylcholine (Vardjan et al., 2007). These results are consistent with the view that fusion-pores, when they are established, are relatively stable structures (Jorgačevski et al., 2010). The regulation of exocytotic release of hormones and transmitters, thus involves also the regulation at the fusion-pore level, at the level, when the fusion-pore has been already established, but is too narrow to functionally contribute to the exit of secretions form the vesicle lumen. In the present paper we have revealed that fusion-pores may exhibit distinct fusion-pore diameters and the future work will have to address question of how these open fusion-states transit to a release productive state.

### Conflict of interest statement

The authors declare that the research was conducted in the absence of any commercial or financial relationships that could be construed as a potential conflict of interest.

## References

[B1] Alvarez de Toledo G. Fernandez-Chacon R. Fernandez J. (1993). Release of secretory products during transient vesicle fusion. Nature 363, 554–558 10.1038/363554a0 8505984

[B2a] Ben-Tabou S. Keller E. Nussinovitch I. (1994). Mechanosensitivity of voltage-gated calcium currents in rat anterior pituitary cells. J. Physiol. 476, 29–39 8046633 PMC1160416

[B2] Breckenridge L. J. Almers W. (1987). Currents through the fusion pore that forms during exocytosis of a secretory vesicle. Nature 328, 814–817 10.1038/328814a0 2442614

[B3] Ceccarelli B. Hurlbut W. P. Mauro A. (1972). Depletion of vesicles from frog neuromuscular junctions by prolonged tetanic stimulation. J. Cell Biol. 54, 30–38 10.1083/jcb.54.1.30 4338962 PMC2108853

[B4] Chen Z. Rand R. P. (1997). The influence of cholesterol on phospholipid membrane curvature and bending elasticity. Biophys. J. 73, 267–276 10.1016/S0006-3495(97)78067-6 9199791 PMC1180928

[B5] Chernomordik L. V. Kozlov M. M. (2008). Mechanics of membrane fusion. Nat. Struct. Mol. Biol. 15, 675–683 10.1038/nsmb.1455 18596814 PMC2548310

[B6] Churchward M. A. Rogasevskaia T. Brandman D. M. Khosravani H. Nava P. Atkinson J. K. (2008). Specific lipids supply critical negative spontaneous curvature - an essential component of native Ca2+-triggered membrane fusion. Biophys. J. 94, 3976–3986 10.1529/biophysj.107.123984 18227127 PMC2367177

[B7] Coorssen J. R. Rand R. P. (1990). Effects of cholesterol on the structural transitions induced by diacylglycerol in phosphatidylcholine and phosphatidylethanolamine bilayer systems. Biochem. Cell Biol. 68, 65–69 2350502 10.1139/o90-008

[B8] Henkel A. W. Meiri H. Horstmann H. Lindau M. Almers W. (2000). Rhythmic opening and closing of vesicles during constitutive exo- and endocytosis in chromaffin cells. EMBO J. 19, 84–93 10.1093/emboj/19.1.84 10619847 PMC1171780

[B9] Heuser J. Reese T. (1973). Evidence for recycling of synaptic vesicle membrane during transmitter release at the frog neuromuscular junction. J. Cell Biol. 57, 315–344 10.1083/jcb.57.2.315 4348786 PMC2108984

[B10] Jahn R. Lang T. Südhof T. (2003). Membrane fusion. Cell 112, 519–533 10.1016/S0092-8674(03)00112-0 12600315

[B11] Jesenek D. Perutková S. Kralj-Iglič V. Kralj S. Iglič A. (2012). Exocytotic fusion pore stability and topological defects in the membrane with orientational degree of ordering. Cell Calcium 52, 277–282 10.1016/j.ceca.2012.04.001 22541648

[B12] Jorgačevski J. Fosnaric M. Vardjan N. Stenovec M. Potokar M. Kreft M. (2010). Fusion pore stability of peptidergic vesicles. Mol. Membr. Biol. 27, 65–80 10.3109/09687681003597104 20334578

[B13] Jorgačevski J. Potokar M. Grilc S. Kreft M. Liu W. Barclay J. W. (2011). Munc18-1 tuning of vesicle merger and fusion pore properties. J. Neurosci. 31, 9055–9066 10.1523/JNEUROSCI.0185-11.2011 21677188 PMC6622955

[B12a] Jorgačevski J. Stenovec M. Kreft M. Bajic A. Rituper B. Vardjan N. (2008). Hypotonicity and peptide discharge from a single vesicle. Am. J. Physiol. Cell Physiol. 295, C624–C631 10.1152/ajpcell.00303.2008 18632733 PMC2544434

[B14] Kabaso D. Calejo A. I. Jorgačevski J. Kreft M. Zorec R. Iglič A. (2012). Fusion pore diameter regulation by cations modulating local membrane anisotropy. ScientificWorldJournal 2012:983138 10.1100/2012/983138 22489211 PMC3317573

[B15] Kozlov M. Markin V. (1983). Possible mechanism of membrane fusion. Biofizika 28, 242–247 6849992

[B16] Kreft M. Zorec R. (1997). Cell-attached measurements of attofarad capacitance steps in rat melanotrophs. Pflugers Arch. 434, 212–214 10.1007/s004240050387 9136678

[B17] Lindau M. (1991). Time-resolved capacitance measurements: monitoring exocytosis in single cells. Q. Rev. Biophys. 24, 75–101 2047522 10.1017/s0033583500003279

[B18] Lindau M. Neher E. (1988). Patch-clamp techniques for time-resolved capacitance measurements in single cells. Pflugers Arch. 411, 137–146 3357753 10.1007/BF00582306

[B19] Lollike K. Borregaard N. Lindau M. (1995). The exocytotic fusion pore of small granules has a conductance similar to an ion channel. J. Cell Biol. 129, 99–104 10.1083/jcb.129.1.99 7535305 PMC2120381

[B20] Lollike K. Lindau M. (1999). Membrane capacitance techniques to monitor granule exocytosis in neutrophils. J. Immunol. Methods 232, 111–120 10.1016/S0022-1759(99)00169-6 10618513

[B21] Malarkey E. B. Parpura V. (2011). Temporal characteristics of vesicular fusion in astrocytes: examination of synaptobrevin 2-laden vesicles at single vesicle resolution. J. Physiol. 589, 4271–4300 10.1113/jphysiol.2011.210435 21746780 PMC3180583

[B22] Neher E. Marty A. (1982). Discrete changes of cell membrane capacitance observed under conditions of enhanced secretion in bovine adrenal chromaffin cells. Proc. Natl. Acad. Sci. U.S.A. 79, 6712–6716 6959149 10.1073/pnas.79.21.6712 PMC347199

[B23] Parpura V. Basarsky T. A. Liu F. Jeftinija K. Jeftinija S. Haydon P. G. (1994). Glutamate-mediated astrocyte-neuron signalling. Nature 369, 744–747 10.1038/369744a0 7911978

[B24] Parpura V. Zorec R. (2010). Gliotransmission: exocytotic release from astrocytes. Brain Res. Rev. 63, 83–92 10.1016/j.brainresrev.2009.11.008 19948188 PMC2862866

[B25] Rituper B. Flašker A. Guček A. Chowdhury H. H. Zorec R. (2012). Cholesterol and regulated exocytosis: a requirement for unitary exocytotic events. Cell Calcium 52, 250–258 10.1016/j.ceca.2012.05.009 22726879

[B26] Rosenboom H. Lindau M. (1994). Exo-endocytosis and closing of the fission pore during endocytosis in single pituitary nerve terminals internally perfused with high calcium concentrations. Proc. Natl. Acad. Sci. U.S.A. 91, 5267–5271 8202480 10.1073/pnas.91.12.5267 PMC43975

[B27] Scepek S. Lindau M. (1993). Focal exocytosis by eosinophils-compound exocytosis and cumulative fusion. EMBO J. 12, 1811–1817 8491174 10.1002/j.1460-2075.1993.tb05829.x PMC413400

[B28] Schwartz J. Wilson D. (1992). Preparation and characterization of type 1 astrocytes cultured from adult rat cortex, cerebellum, and striatum. Glia 5, 75–80 10.1002/glia.440050111 1531812

[B30] Stenovec M. Kreft M. Poberaj I. Betz W. Zorec R. (2004). Slow spontaneous secretion from single large dense-core vesicles monitored in neuroendocrine cells. FASEB J. 18, 1270–1272 10.1096/fj.03-1397fje 15180959

[B31] Vardjan N. Stenovec M. Jorgačevski J. Kreft M. Grilc S. Zorec R. (2009). The fusion pore and vesicle cargo discharge modulation. Ann. N.Y. Acad. Sci. 1152, 135–144 10.1111/j.1749-6632.2008.04007.x 19161384

[B32] Vardjan N. Stenovec M. Jorgačevski J. Kreft M. Zorec R. (2007). Subnanometer fusion pores in spontaneous exocytosis of peptidergic vesicles. J. Neurosci. 27, 4737–4746 10.1523/JNEUROSCI.0351-07.2007 17460086 PMC6672992

[B33] White J. (1992). Membrane fusion. Science 258, 917–924 10.1126/science.1439803 1439803

